# Low-dose methotrexate-induced renal failure in a patient with ectopic pregnancy: a case report

**DOI:** 10.1186/s13256-023-03834-z

**Published:** 2023-04-02

**Authors:** Lili Zhang, Chen Liu, Ling Xiao, Yun Liu

**Affiliations:** Department of Obstetrics and Gynecology, Beijing Friendship Hospital, Beijing, China

**Keywords:** Methotrexate, Ectopic pregnancy, Renal failure, Toxicity

## Abstract

**Background:**

Methotrexate is an anticancer drug from the antimetabolite class. It is also used in gynecology and obstetrics for the medical treatment of ectopic pregnancies. Low-dose methotrexate-induced adverse toxic effects are rare. We report a case of toxic effect associated with severe renal insufficiency induced by LD-MTX (Low-Dose Methotrexate) for ectopic pregnancy.

**Case presentation:**

A 46-year-old Chinese woman was in an operation for an ectopic pregnancy of tubal interstitial pregnancy. The embryo villus was so little that we were not sure if it was evacuated, then it was followed with 50 mg methotrexate injection of intramuscular adjacent the uterine horn in the operation. 48 hour later after injection the patient presented with renal failure. The personalized genetic testing showed that MTHFR (677C > T) and ABCB1 (3435T > C) were detected. Gradually, the symptoms improved after calcium leucovorin (CF) rescue, continuous renal replacement therapy (CRRT), promoting blood system regeneration, and multiple supportive treatments.

**Conclusions:**

When toxic effects are suspected, detecting the polymorphisms of an MTHFR gene and monitoring MTX concentration in blood could assist us to formulate individualized and active treatments. The management should be multidisciplinary and as much as possible within an intensive care unit.

## Introduction

Methotrexate (MTX), a folic acid antagonist, is widely used in the treatment of tumor chemotherapy, Rheumatoid arthritis, psoriasis *et al.* It is also an effective treatment for early unruptured ectopic pregnancy (EP). Treatment with MTX, highly toxic to rapidly replicating tissues, achieves results comparable to surgery for the treatment of appropriately selected ectopic pregnancies and is used commonly [1]. High-dose methotrexate (HD-MTX) is commonly defined as an intravenous dose greater than 500 mg/m^2^ [2]. HD-MTX is recommended as an essential component of chemotherapy for acute lymphoblastic leukaemia in clinical guidelines [3]. With strict criteria of inclusion and follow-up, single-dose intramuscular methotrexate (50 mg/m^2^) is a successful method for the treatment of selected cases of ectopic pregnancy [4]. It is regarded as LD-MTX. The primary toxic effects of LD-MTX may only be limited to nausea, vomiting, and diarrhea, which are common adverse reactions after chemotherapy and are often ignored. To date, eight cases of single-dose LD-MTX protocols that induced serious adverse toxic effects on patients with EP have been reported [5]. 7 studies also summarize case series, and case reports regarding low-dose methotrexate toxicity in other disease [6].

Methylene tetrahydrofolate reductase (MTHFR) is an important enzyme in the MTX pathway and is involved in folate metabolism and DNA synthesis. Several studies have examined the association between the MTHFR C677T polymorphism and MTX toxicity and efficacy, but their conclusions remain controversial. A review study indicated that the MTHFR C677T polymorphism could be used as a predictor of MTX toxicity in RA patients [7]. A meta-analysis included 17 studies showed that no significant correlation between MTHFR C677T/A1298C genetic polymorphism and patients' toxicity or the relapse and survival associated with MTX chemotherapy. But a tendency toward increased risk of hepatotoxicity was also present for acute lymphoblastic leukemia in the mutation model [8]. We report a case of ectopic pregnancy with MTHFR gene mutations associated with severe renal failure induced by LD-MTX. In this case, the failure of renal function returned 1 month later.

## Case presentation

A 46 years old Chinese woman, gravida 4 para 1 abortion 2, was admitted to the Department of Obstetrics and Gynecology of the Beijing Friendship Hospital (Beijing, China) on July 14, 2020, with complaints of “amenorrhea for 6 weeks, ectopic pregnancy was diagnosed by ultrasound”. The patient was presented elevated beta-human chorionic gonadotropin (β-hCG) level (4137.60 mIU/L), and ultrasound findings of a mixed echogenic mass of 0.6 × 0.5 × 0.6 cm in the left cornual uterus. Magnetic resonance imaging (MRI) scans showed cystic signal of 0.5 cm × 0.6 cm under the left cornua of the uterus. She was diagnosed with ectopic pregnancy (interstitial tubal pregnancy or cornual pregnancy). 2 days later the patient had a laparoscopic surgery with cornual uteri resection and left salpingectomy. According to Chinese consensus recommendations, If incomplete excision of the ectopic pregnancy is a concern, prophylactic intramuscular treatment with a single dose of MTX may be considered and may significantly reduce the rate of persistent ectopic pregnancy (recommendation grade C), as the villi was incomplete and then it was followed with an intramuscular injection adjacent the uterine horn of MTX at a total dose of 50 mg (< 1 mg/kg). There was no immediate incident.

On day 1 after MTX administration (“day 1” was abbreviated as “D1”), She began to nausea, vomit, and diarrhea for 3 times. The serum creatine (Cr) increased to 178.6 × μmol/L (Table 1). Considering of the increasing Cr, we analysised the most likely causes: one is the adverse reaction of laparoscopic pneumoperitoneum; The other maybe drug factor. The MRI was enhanced and the contrast medium Omniscan was used before the operation which has side effects of renal function injury. MTX may also induce gastrointestinal reaction, but the renal function injury by MTX cannot be entirely excluded. So we gave her intravenous fluids 2000 ml, and Cr was measured again the next day. On D1 β-hCG level fell by half to 1778 mIU/L, so the treatment for EP is succeed.

**Table 1:** Laboratory data in relation to LD-MTX administration

Date	WBC (10^9^/L)	RBC (10^12^/L)	HGB (g/L)	PLT (10^9^/L)	ANC (%)	Cr μmol/L	Urea mmol/l	HCG mM /L)	MTX
2020/7/14						57.2		4137.6	
2020/7/15	5.27	4.43	135	232	57			4026.7	
2020/7/16								4726	
2020/7/17(D1)	10.53	4.09	123	197	84.4	178.6		1778	
2020/7/18(D2)	8.29	3.89	116	170	90.5	329.1	8.32		
2020/7/19(D3)	7.16	3.39	102	156	92.8	387.6	9.61		
2020/7/20(D4)	5.25	3.29	98	145	90.9	456.2	13.19	331.53	
2020/7/21(D5)	2.85	3.23	95	131	72.4	370.3	9.91		
2020/7/22(D6)	2.78	3.46	101	147	60.2	332.3	8.6		
2020/7/23(D7)	2.9	3.41	103	146	68.6	305.7	6.47		0.262
2020/7/24(D8)	4.46	3.1	95	125	75.3	274	4.78	86.35	
2020/7/25(D9)	4.3	3.15	96	155	74.2	270.6	4.81		
2020/7/26(D10)	4.49	3.25	101	206	73.7	266.1	5.05		
2020/7/27(D11)	4.08	3.52	104	279	67.9	255.4	4.5		0.116
2020/7/28(D12)	3.53	3.1	95	312	73.9	239.8	4.73		
2020/7/29(D13)	3.49	3.24	96	358	66.4	229.8	4.76		
2020/7/30(D14)	3.31	3.25	99	412	59.5	213.2	5.28		0.06
2020/7/31(D15)	2.78	3.32	98	434	49.3	208.2	6.63	13.1	
2020/8/1(D16)	2.53	3.26	98	434	41	217.7	9.67		
2020/8/2(D17)	2.64	3.56	105	497	37.9	202.2	9.82		
2020/8/3(D18)	2.64	3.33	99	382	38.1	176.2	9.53		
2020/8/4(D19)	3.42	3.61	108	417	40.4	163.9	11.18		
2020/8/5(D20)	3.27	3.59	108	400	49.9	154.9	10.79		
2020/8/6(D21)	3.83	3.57	107	366	53	136.5	10.04		
2020/8/7(D22)	4.46	3.68	111	333	56.7	139.3	9.08	3.57	
2020/8/10	5.76	3.72	112	296	65.1	118.4	8.92		
2020/8/12	6.47	3.88	114	331	70.8	107.8	8.06		
2020/8/14	6.33	3.53	105	254	69.3	96.7	6.35		
2020/8/18	5.78	3.4	104	245	63.6	91.3	6.03		
2020/8/21						87	4.58		
2020/11/5	4.83	4.61	135	258	58.8	66.1	5.63		
2021/7/14						62.6	5.35		

On D2, She developed oliguria and lower limbs edma. The Cr increased to 329.1 × μmol/L. She also diarrheaed 9 times a day. An immediate urological ultrasound showed enlargement of the right kidney and enhanced echo of both kidneys. The diagnosis of renal failure caused by MTX is clear. MTX retention was treated by initiating calcium leucovorin (CF) rescue, which was started since D2 (150 mg, q3h, IV), and the dose was decreased on D5 (30 mg, q8h, IV) due to the abnormal MTX concentration. Sodium bicarbonate was administered intravenously to alkalize urine. Furosemide was used because of oliguria.

On day 4(D4), continuous renal replacement therapy (CRRT) was performed from D4 to D14. Although CRRT was given, therapeutic drug monitoring showed that the serum MTX concentrations were 0.262 µmol/L (D7) and 0.116 µmol/L (D11) (in Table 1), which were significantly higher than the normal level. The personalized genetic testing showed that MTHFR TT (677C > T) and ABCB1 (3435T > C) were detected, indicating a 66% decrease in the MTHFR activity. For the patient with genetic abnormality the generation of folic acid was seriously prohibited, which resulted in the decrease of MTX clearance. After the CRRT and CF rescue, the serum MTX concentrations is decreasing.

**Fig. 1:**
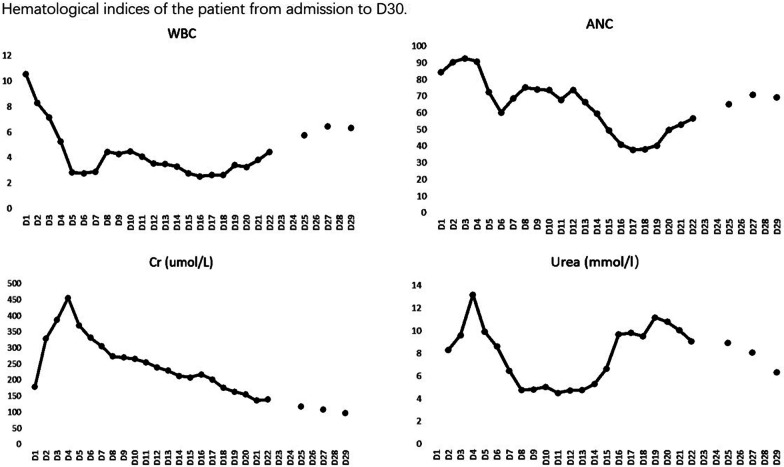
Hematological indices of the patient from admission to D30

On D5 (Fig. 1), the WBC and ANC began to increase and On D 22 descended to the normal level. On D22, the Cr began to return to normal gradually. On D15, the β-HCG level descended to the normal level. It was monitored until negative reports twice; The Urea began to increase on D1 and began to decrease on D4 after the CRRT. When the CRRT stopped, it had a slight increase and On D20 it began to return to normal again. The kidney ultrasound was carried out again and showed no obvious abnormalities. To date, we have followed up her for 24 months, and her general condition is satisfactory.

## Discussion

This is a healthy middle-aged woman without liver or kidney disease. Severe renal impairment occurred after the treatment of low doses of MTX of 50 mg during the operation. One day after MTX administration, nausea, vomiting, and diarrhea appeared. On the second day she was aggravated with oliguria and renal impairment. After treatment with CF, hydration, alkalization of urine, and CRRT, her indicators of renal function returned to normal 1 month later. And after 1 year of follow-up, the patient get right。

Overall, MTX is a safe and effective treatment for an unruptured ectopic pregnancy. Low-dose MTX (LDMTX) has been widely used to avoid the necessity of undergoing surgery and preserve the integrity of the fallopian tube [6, 9, 10]. Very rarely, life-threatening complications have been reported with MTX. To date, only 9 cases of single-dose LD-MTX protocols that induced serious adverse toxic effects on patients with EP have been reported [5]. In the 10 cases including ours, all the patients had myelosuppression [11–18] most of them had nausea and vomiting, All the 9 patients had fever except our case. 2 patients had septic shock [11, 14], 3 patients had liver injury [14, 15, 18], 2 patients including our case had renal failure [18] and 3 patients died [14, 15, 18]. In the majority of cases, the patients began to recover in the period of 14–17 days [5].

In our case, the maximum reduction in the number of leukocytes was estimated to occur approximately 5 days after the last administration of MTX, and the count recovery achieved 22 days later. The renal failure occurred on the next day after MTX, and also recovered 22 days later. Her most serious complication is renal failure, the other patient who also had renal failure died without CRRT and CF rescue [18].

The MTX undergoes additionally a bidirectional transport within the renal tubule, an active secretory process utilising the general organic acid transport mechanism, and an active reabsorption process unaffected by acidic compounds [19]. As the elimination half life of MTX increases with the severity of renal impairment. The decrease of the total clearance of MTX is directly related to a decrease in renal function [20]. Therefore, when patients experience adverse reactions of methotrexate to renal function damage, accumulation of methotrexate will be more serious. Then the treatment mainly depends on hemodialysis, especially high-throughput hemodialysis because of the low molecular weight of methotrexate, which make the condition more serious. In general, a sustained elevation of plasma MTX concentrations at 24 hour (> 5–10 µM), 48 hour (> 1.0 µM), and 72 hour (> 0.1 µM) after administration of MTX are predictive for the development of toxicity [21]. The laboratory results showed that the MTX concentrations were higher. On D7 (Table 1), the MTX concentration is 0.262 µM, On D11, it went down to 0.116 µM. On D14, it was normal of 0.06 µM and the CRRT stopped. The CRRT lasted totally for 11 days.

High-dose methotrexate is used for a range of cancers. Although HDMTX is safely administered to most patients, it can cause significant toxicity, including acute kidney injury, attributable to crystallization of methotrexate in the renal tubular lumen, leading to tubular toxicity [2]. In this case, severe renal failure occurred after low dose administration. It’s really rare. Genetic testing revealed that the patient had a genetic mutation. It indicated that MTHFR (677C > T), the activity of methylene tetrahydrofolate reductase was decreased according to the genotype of the locus. And the report ABCB1 (3435T > C) mutation heterozygous type showed high risk of hepatotoxicity and hematotoxicity. C677T is one of the most common single nucleotide polymorphism in MTHFR gene. Enzyme activity is reduced by 30% and 65%, respectively [22]. Interestingly, their results showed that CC carriers had a lower success rate than CT and TT carriers by first MTX injection and another dosage of MTX treatment in genotypes CT and TT carriers [23]. However, genetic testing is generally not used as an essential examination due to the high cost and the long procedure. For patients with significantly fast adverse drug reactions on MTX, the possibility of genetic abnormalities should be warned and the test should be done.

This patient’s renal function recovered on D22. After MTX injection the patient developed gastrointestinal symptoms first. Although the way of the first CF rescue varies with different tumor types and doses, for the 24 hour infusion regimen, it was recommend administering the first dose of leucovorin at 36–44 hour after the start of HD-MTX infusion. When serious adverse reactions occur, the timing of the first dose should be adjusted individually. The leucovorin should be administered until the MTX concentration is below 0.1–0.2 μmol/L [24]. Although the guideline is for HD-MTX infusion, when the patient’s MTHFR gene had the polymorphisms mutation, it can provide guidance for us. For patients without normal MTX clearance, the rescue dose of CF needs to be appropriately increased to ensure the efficacy.

Although the adverse reactions caused by low-dose methotrexate in this case were confused, the patient improved eventually 22 days later, which was similar to the reported case [5]. Therefore, active treatment after the occurrence of adverse reactions has a relatively good prognosis.

The lesson from this case is that low doses of methotrexate may also cause serious adverse reactions, especially in patients with genetic mutations. Due to the low incidence and high price, routine genetic testing before methotrexate injection cannot be done. But when toxic effects such as overlooked gastrointestinal, skin, and mucosal symptoms occurred, methotrexate adverse reactions should be considered, and the following active treatment will have a better outcome. And when renal failure occurred, the CRRT should be considered.

## Conclusion

Although MTX is a very useful drug in the treatment of EP, when used in the lowest dose, it may prove to be fatal. Routine CBC, liver, and renal function tests for patients on MTX should be performed at more frequent. When toxic effects are suspected, detecting the polymorphisms of MTHFR gene and monitoring MTX concentration could assist us to formulate individualized and active treatments. After aggressive and effective treatments, most of them recovered nearly a month later.

## Data Availability

Not applicable.

## Abbreviations

ADRs: Adverse drug reactions
ANC: Absolute neutrophils count
CRRT: Continuous renal replacement therapy
Cr: Creatinine
CF: Calcium leucovorin
EP: Ectopic pregnancy
LD-MTX: Low-dose methotrexate
MTX: Methotrexate
MTHFR: Methylene tetrahydrofolate reductase
WBC: White blood cell count
